# Bronchial Epithelial Cells from Asthmatic Patients Display Less Functional HLA-G Isoform Expression

**DOI:** 10.3389/fimmu.2017.00006

**Published:** 2017-01-23

**Authors:** Federico Carlini, Christophe Picard, Céline Garulli, David Piquemal, Pierre Roubertoux, Jacques Chiaroni, Pascal Chanez, Delphine Gras, Julie Di Cristofaro

**Affiliations:** ^1^Etablissement Français du Sang Alpes Méditerranée, Marseille, France; ^2^Aix-Marseille Univ, CNRS, EFS, ADES, “Biologie des Groupes Sanguins”, Marseille, France; ^3^Aix-Marseille Univ, INSERM U1067 CNRS UMR 7333, Marseille, France; ^4^ACOBIOM, Montpellier, France; ^5^INSERM U491, Génétique Médicale et Développement, Aix-Marseille Université, Marseille, France; ^6^Clinique des Bronches, Allergie et Sommeil, AP-HM Hôpital Nord, Marseille, France

**Keywords:** asthma, epithelium, HLA-G, immunomodulation, isoforms

## Abstract

Not all asthmatic patients adequately respond to current available treatments, such as inhaled corticosteroids or omalizumab^®^. New treatments will aim to target the bronchial epithelium–immune response interaction using different pathways. HLA-G is involved in immunomodulation and may promote epithelial cell differentiation and proliferation. HLA-G protein has several isoforms generated by alternative splicing that might have differential functionalities. HLA-G protein expression and genetic polymorphisms have been reported to be associated with asthma. Our hypothesis is that bronchial epithelium from asthmatic patients displays less functional HLA-G isoforms. HLA-G transcriptional isoforms were quantified by real-time PCR in human bronchial epithelium cells (HBEC) grown in air–liquid interface culture obtained from five healthy controls (HC), seven patients with mild asthma (MA), and seven patients with severe asthma (SA). They were re-differentiated, and IL-13 exposure was used as a proxy for a pro-inflammatory cytokine. HLA-G protein expression was assessed by western blot analysis. HLA-G allele was typed by direct sequencing. Our results showed that both MA and SA display less functional HLA-G isoforms than HC (*p* < 0.05); *in vitro* HBEC re-differentiation from SA displays a particular isoform expression profile compared to MA and HC (*p* = 0.03); HLA-G*01:06 frequency in MA and SA was significantly higher than in the healthy population (*p* = 0.03 and *p* < 0.001, respectively); and IL-13 exposure had no impact on HLA-G expression. Our results support that an impaired expression of HLA-G isoforms in asthmatic patients could contribute to the loss of inflammation control and epithelium structural remodeling. Therefore, HLA-G might be an interesting alternative target for asthmatic patients not adequately responding to current drugs.

## Introduction

Asthma affects almost 300 million people worldwide and is related to a chronic inflammation of the airways. It groups various phenotypes which can be classified according to clinical severity, age of onset, and different immune responses, i.e., T2-associated and non-T2 asthma (1). T2-associated asthma is characterized by the release of interleukins IL-13 and IL-5, promoting eosinophilic infiltration, pro-inflammatory loops, epithelial cell proliferation, goblet-cell metaplasia, and ciliary beating alterations (1). Non-T2 asthma is more often associated with neutrophilic inflammation and a mixed T1 and T17 cytokine milieu (2).

The bronchial epithelium is abnormal in asthma, with structural changes and thickening of the sub-epithelial layer (3), and its function is characterized by an exaggerated release of various cytokines, including TSLP, IL-33, and IL-25, as well as impaired inflammation resolution (4). Human bronchial epithelium cells (HBEC), in direct contact with the external environment, are key sensors of viruses, allergens, or pollutants through pathogen-associated molecular patterns (PAMPs), NOD2, and TLR [for review, see Ref. (5)]. HBEC can initiate and perpetuate immune responses by recruiting effector cells through the release of mediators (2). HBEC from severe asthma patients secrete higher levels of IL-8, a chemoattractant for neutrophils, and reduced levels of lipoxins, mediators which normally resolve inflammation (6).

Therapeutics based on the association of corticosteroids, leukotriene receptor antagonists, and long-acting beta agonists are efficient in about 80% of asthmatic patients. Immune-based therapies have been developed to specifically reduce T2 immune cell activity either by blocking the pro-inflammatory cytokine loop (using, for example, anti-IL-5, IL-13, or IL-4, monoclonal antibodies) or by blocking activating-receptor fixation (using, for example, anti-IgE monoclonal antibody preventing IgE fixation to FcεRI, omalizumab^®^), significantly increasing various outcomes, including exacerbation rates, and improving pulmonary function tests in patients with T2-associated asthma not responding to inhaled corticosteroid treatment (ICS) (7).

Despite these approaches, there is no drug at present which interferes with the natural history of the disease and can be called a disease modifier. Thus, efforts are made to develop new therapeutic strategies, and in this regard, the interaction between the bronchial epithelium and immune response is gaining interest (8, 9).

Besides their role in promoting inflammation, HBEC also express the immunotolerant HLA-G molecule, extensively documented for its involvement in immune scenarios, such as solid tumors, viral infections, and autoimmune diseases [for review, see Ref. (10)].

In asthma, HLA-G expression was found to be increased in lung tissues. Broncho alveolar lavage fluid and plasma HLA-G levels were higher in asthmatic individuals than in healthy volunteers (11–13). Interestingly, White et al. reported that HLA-G expression in healthy HBEC was independent from T2-related cytokines IL-4, IL-5, or IL-13 (14).

HLA-G inhibits NK- and cytotoxic T lymphocyte-mediated activity as well as B cell activation *via* their inhibitory receptor (ILT-2, -4 and KIR2DL4). This non-classical HLA class I molecule also acts indirectly on immune control as HLA-E preferentially loads its signal peptide (15).

HLA-G is involved in epithelial cell differentiation: its expression increases proliferation and differentiation properties of amnion epithelial cells (16), and it is upregulated in fetal lungs suggesting a role in lung development (17).

Functional analysis of HLA-G is challenging because of its many levels of polymorphism: dimerization, association with β2-microglobulin, and alternative splicing (18). Primary HLA-G mRNA splicing generates both membrane-bound (HLA-G1–4) and soluble (HLA-G5–7) isoforms (18), and membrane-bound isoforms can also be shed *via* proteolytic cleavage by matrix metalloproteinase-2 (19). The HLA-G1 and -G5 isoforms display a similar structure to HLA class Ia molecules (α1, α2, and α3 domains), whereas HLA-G2 and -G6 are composed of α1 and α3 domains, -G3 and -G7 contain an α1 domain, and -G4 is composed of α1 and α2 domains (18). These truncated isoforms appear to be differently processed, as HLA-G2, -G3, and -G4 are sensitive to endoglycosidase H, suggesting a non-involvement of the Golgi apparatus, conversely to HLA-G1 and classical HLA class I (20).

Alpha1 and α3 domains have been, respectively, described to bind the KIR2DL4 inhibitory receptor and ILT-2 and -4 (21, 22). Thus, differential functional activity can be expected for these isoforms. Both HLA-G1 and -G5 isoforms reduce NK cell cytotoxicity with an additive effect (23). Two studies concluded functional activity for HLA-G2, -G4, and -G6 (20), whereas conflicting results were published concerning HLA-G3 (20, 24). A supplementary level of splicing is generated due to the 14 bp insertion polymorphism in exon 8 (ex8); this insertion introduces an additional splice site which removes the first 92 bp of exon 8, generating a more stable transcript and a protein that inhibits NK cytotoxicity more efficiently (25, 26).

Several studies focused on HLA-G isoform expression supported a differential impact on their immunomodulation and differentiation properties. In pregnancy, HLA-G5 mRNA and protein was significantly lower in abortion-threatened women, whereas HLA-G7 showed no significant variation in peripheral blood mononuclear cells (27). HLA-G1, -G2, -G5, and -G6 expression varied during embryonic maturation whereas HLA-G3 and -G4 displayed no variation in preimplantation embryos during *in vitro* differentiation (28). In normal HBEC, HLA-G1 and -G5 transcripts are both expressed, with less HLA-G1 than HLA-G5 protein (14). Conversely, in lung cancer, the transcriptional level of HLA-G5 is lower than HLA-G1, with a higher overall HLA-G mRNA and protein isoform expression in tumor tissues compared to unaffected lung samples (29).

These data suggest that the bronchial epithelium may play a central role in pulmonary local immune responses *via* HLA-G expression, in an independent manner from pro-inflammatory cytokine regulation. HLA-G expression could also influence HBEC proliferative and differentiation abilities. A dysregulation of HLA-G isoforms in an asthma context could lead to both an impaired immunoregulatory capacity and altered epithelium differentiation properties.

The main objectives of this study were to analyze HLA-G isoform expression in normal, severe, and mild asthmatic HBEC, during the HBEC re-differentiation process, and to investigate HLA-G regulation by a T2-related representative cytokine (IL-13) activation pathway. This study, performed on a rarely accessible method of HBEC derived from endobronchial biopsies fully re-differentiated into bronchial epithelium tissue using an air–liquid interface (ALI) culture, is the first to have investigated into the expression of all of the HLA-G mRNA isoforms.

## Materials and Methods

### ALI Culture of Human Bronchial Epithelial Cells

Healthy control (HC) subjects and patients with mild asthma (MA) and severe asthma (SA) were recruited after written informed consent at the Clinique des Bronches, Allergie et Sommeil, AP-HM Hôpital Nord, Marseille, France. The patients belonged to the Bronchial Obstruction and Asthma Cohort (COBRA—*Cohorte Obstruction Bronchique et Asthme)* sponsored by the French National Institute of Health and Medical Research, INSERM [IDRGB 2008-A00284-51, Afssaps 2008-0113]. Asthma diagnosis and severity classification were carried out as previously described (6). Patients’ characteristics are detailed in Table 1.

**Table 1:** **Patient characteristics and their differences**.

Patients’ characteristics	Healthy controls	MA	SA	*p*-Value
Number under study	15	16	16	
Age (years) (medians and range)	46 (17–61)	60 (36–65)	59 (47–71)	NS
Sex (% female)	80	71.4	57.1	NS
Control of asthma (%)	NA	100	14	0.002
Exacerbations (number during last year)	NA	14	100	0.002
Best FEV1 (% and predicted)	100 (100–122)	100 (76.4–109)	70 (60.4–85)	0.01
Blood eosinophils/mm^3^ (medians and range)	150 (130–200)	110 (36–190)	180 (40–1,290)	NS
Allergy (%)	0	28.6	57.1	NS
Sinusitis (%)	40	71.4	85.7	NS
Bronchorrhea (%)	0	42.8	57.1	NS
GERD (%)	40	42.8	57.1	NS
Comorbid condition, ≥1 (%)	20	42.8	57.1	NS
ICS (%)	NA	42.8	100	0.03
LAβ2 (%)	NA	42.8	100	0.03
SAβ2 (%)	NA	71.4	100	NS
OCS (%)	NA	0	57.1	0.01

*Differences between two groups were analyzed using the Mann–Whitney U test, and differences among the three groups were analyzed using the Kruskal–Wallis test, followed by a post hoc Bonferonni analysis. The χ^2^ test was used to evaluate dichotomous characteristics of patients*.

*NA, non-applicable; NS, non-significant*.

Human bronchial epithelium cells obtained from bronchial biopsy specimens were cultivated under ALI conditions, as previously described (6). Briefly, bronchial epithelial biopsy tissue was dissociated and suspended in bronchial epithelial growth medium (Lonza, Switzerland). After seeding in multiwell plates coated with a solution of fibronectin and collagen, cells were expanded in a flask and then plated on uncoated nucleopore membranes in a 1:1 mixture of bronchial epithelial growth medium and Dulbecco’s modified Eagle medium (Life Technologies, France) applied on the basal side only to establish the ALI. Cells were maintained in culture for 21 days to obtain a differentiated cell population with a mucociliary phenotype.

### Cell Line Culture

Human choriocarcinoma cell line (JEG-3) was used as control for HLA-G expression. JEG-3 (HTB-36) cells were obtained from the American Type Culture Collection (LGC Standards, France). All reagents and culture media were provided by Life Technologies. JEG-3 cells were cultured in DMEM containing 10% fetal bovine serum.

### RNA Extraction and Reverse Transcription

Expression studies according to asthmatic severity, re-differentiation, and IL-13 stimulation were performed on HBEC RNA from 19 patients (HC *N* = 5, MA *N* = 7, SA *N* = 7) at day (D) 0, D7, D14, and D21, with and without IL-13 stimulation (5 and 10 ng, 213-ILB/CF; R&D systems, France). HBEC total RNA was isolated using the RNeasy kit (Qiagen, France).

Total RNA from JEG-3, at 60% confluence after 24 h, was extracted from cell lysis using the mirVana™ PARIS™ Kit (Life Technologies).

cDNA was reverse transcripted using Superscript III Reverse Transcriptase (Life Technologies) following manufacturer’s recommendations.

### Real-time PCR Analyses

Real-time PCR analyses were performed using TaqMan technology (Life Technologies) using nine primer/probe targeting: all HLA-G transcripts (ex1–2 and ex5–6), HLA-G1/4/5 (ex2–3), HLA-G1/5 (ex3–4), HLA-G2/6 (ex2–4), HLA-G3 (ex2–5), HLA-G5/6 (int4–ex5), and ex8 14 bp ins/del polymorphism. All were designed by Acobiom (Montpellier, France) except pan HLA-G ex5–6 (Hs03045108_m1, Life Technologies) (Table S1 and Figure S1 in Supplementary Material). Actinβ (ACTB) was used as an endogenous control (normalizer) and CCL26 (eotaxin-3) as an IL-13 stimulation control (ACTB Hs99999903_m1; CCL26 Hs00171146_m1; Life Technologies).

Different plasmids were constructed to check specificities and efficiencies of primers/probes used for real-time PCR analyses. One ACTB and eight HLA-G amplicons were obtained from PCR performed on JEG-3 or HBEC cDNA using the Taq DNA Polymerase recombinant kit (Life Technologies) according to manufacturer’s protocol (Table S2 in Supplementary Material for plasmids characteristics). Purified PCR amplicons (NucleoSpin, Macherey-Nagel, France) were cloned in pGEM-T vectors (Promega, France) and One Shot TOP10 Competent Cells (Life Technologies) as previously described (30). Efficiencies of HLA-G and ACTB primer/probe were calculated using plasmid dilutions (first point at 10^6^ copie numbers/μl and six points dilution 1:10), and checked in JEG-3 dilutions, according to MIQE Guidelines (31).

Real-time PCR mixture was performed in a total volume of 20 µL, including 3 µL of cDNA or plasmid dilution, 1× TaqMan^®^ Fast Universal PCR Master Mix no AmpErase^®^ UNG (Life Technologies), 900 nM of each primer, and 200 nM of probe. StepOnePlus™ Real-Time PCR System (Life Technologies) was used to perform real-time PCR according to manufacturer’s recommendations (95°C for 20 s, followed by 40 cycles of 95°C for 1 s, and 60°C for 20 s). Each experiment was carried out in duplicate, including a negative control, a positive control, and appropriate plasmid dilutions.

Cycle threshold (Ct) data were determined with defined threshold settings [0.2 for ACTB and 0.1 for CCL26 (eotaxin-3) and HLA-G isoforms]. Average Ct was calculated with StepOne 2.1 software (Life Technologies), excluding Ct duplicates with an SD higher than 0.5.

JEG-3 and an HBEC HC sample were respectively used as calibrators for HLA-G isoforms quantification and exon 8 14 bp ins/del. Each D0 sample from the re-differentiation experiment was used as a calibrator to quantify HLA-G isoforms from D7 to D21. Each unstimulated HBEC sample from the IL-13 stimulation experiment (5 and 10 ng of IL-13) was used as a calibrator. ACTB was used as normalizer.

Quantification was carried out using the relative standard curve method with the following equation (32): Fold change = (*E target*) × ΔCt *target*/(*E normalizer*) × ΔCt *normalizer, w*ith, *E* = 10^[−1/slope]^, efficiency calculated from standard curve (plasmid dilution), ΔCt *target* = Ct *target*^calibrator^ − Ct *target*^sample^ and ΔCt *normalizer* = Ct *normalizer*^calibrator^ − Ct *normalizer*^sample^.

HLA-G isoforms ratio was calculated according Pan HLA-G ex5–6 expression using the following equation (33): Ratio Gx = 2^−^Δ^Ct Gx^/2^−ΔCt Pan HLA-G ex5–6^ with Gx being the HLA-G isoform understudy.

### HLA-G Protein Expression

The 4H84 antibody was used to confirm protein expression in HBEC. 4H84 is directed against a linear epitope of the unfolded α1 domain of the HLA-G free protein, which is present in all HLA-G isoforms (34, 35). Western blot analyses were performed on HBEC protein lysates at D21 (HC *N* = 10, MA *N* = 9, SA *N* = 9). Cells were rinsed with PBS and extracted in lysis buffer (50 mM Tris–HCl, pH 7.6, 150 mM NaCl, 1 mM EDTA, 1% Triton X-100, 0.1% sodium dodecyl sulfate, 2 mM MgCl_2_) containing a protease inhibitor cocktail (Sigma-Aldrich, France). Samples were normalized for protein content with the BCA protein assay kit (Perbio Science, France). Protein samples (20 µg) were fractionated on a 4–15% SDS-PAGE, followed by a semi-dry transfer onto a PVDF membrane (Biorad, France). Blots were blocked with blotting buffer (5% non-fat milk, 10 mM Tris–HCl, 150 mM NaCl, 0.1% Tween 20) before being probed with mouse monoclonal antibody against HLA-G (1:500) (clone 4H84; Perbio Science, France). Rabbit polyclonal antibody against glyceraldehyde 3-phosphate dehydrogenase (GAPDH) (1:200) (clone 9545; Sigma-Aldrich) was use for standardization. Horseradish peroxidase-conjugated anti-mouse IgG (1:20,000, SantaCruz) were used as secondary probes. The densitometric analysis of immunoblots was performed using Alphaview Software (ProteinSimple). Results are expressed as the ratio of HLA-G/GAPDH bands mean intensity (A.U., arbitrary unit).

### HLA-G Allele Typing

Allele typing was carried out by PCR and direct sequencing from all HBEC cDNA using JEG-3 cDNA as control (G*01:01; G*01:01, exon8 14 bp ins/ins) (18). PCR amplification was performed using a Multiplex PCR Kit (Qiagen) following manufacturer’s recommendations with primers targeting HLA-G exon 1 to exon 5 (Table S1 in Supplementary Material). Direct sequencing was performed using Big Dye Terminator V1.1 kit (Life Technologies) according to the manufacturer’s protocol. Alignments were performed with Codon Code Aligner version 3.7.1 (Codon Code Corporation, USA) using HLA-G allele sequences from the official IMGT/HLA database 3.20.0 May 2015 (36).

### Statistical Analyses

All association and correlation tests were performed with GRAPH PAD Prism 5 software (CA, USA, www.graphpad.com). Differences among patient characteristics were tested using Mann–Whitney *t*-test to test two modalities. Kruskal–Wallis one-way ANOVA followed by Dunn *post hoc* test was used to test more than two modalities.

mRNA expression fold change data are presented as median and range (min, max). Correlations between HLA-G isoforms at D0, D7, D14, and D21 for each clinical condition (HC, MA, and SA) were performed using Pearson’s chi-square test. HLA-G isoform data that displayed a significant correlation were cumulated in subsequent analyses. HLA-G isoform data that displayed no correlation were analyzed separately.

Associations and correlations between HLA-G isoform expression and clinical (allergy, asthma severity, eosinophil count), experimental (HBEC re-differentiation, IL-13 stimulation), biological (age, gender), and genetic parameters were done using the Mann–Whitney *t*-test and Kruskal–Wallis one-way ANOVA. Pearson’s test was used for numerical correlation. To further investigate the effect of HLA-G alleles on HLA-G isoform mRNA expression, the comparison of a single observation to the mean of the sample has been used (37). In this particular case, data are represented as mean with SD. Repeated-measures one-way ANOVA was applied specifically to test the association between HLA-G isoform mRNA expression and experimental parameters.

## Results

The characteristics of the individuals studied are shown in Table 1.

### HLA-G Isoforms Expression in HBEC from HCs

**Figure 1:**
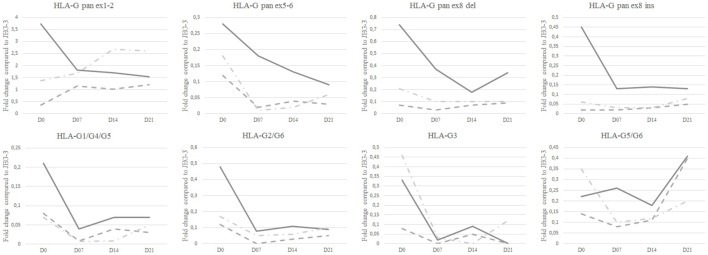
**HLA-G isoform transcriptional expression analyzed by Q-PCR**. Median fold change as compared to JEG-3 in healthy controls (solid line), patients with mild asthma (dashed line), and patients with severe asthma (dashed and dotted line) at day 0 (D0), D7, D14, and D21. Medians with range are described in Table S3 in Supplementary Material.

All HLA-G mRNA isoforms were observed in re-differentiated HC epithelium at D21 except HLA-G3 (Table S3 in Supplementary Material). Compared to JEG-3, extensively analyzed for HLA-G expression, HBEC showed a lower expression of all HLA-G isoforms, except pan ex1–2 HLA-G (Figure 1).

**Figure 2:**
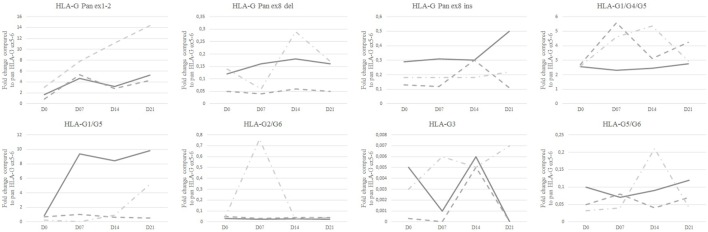
**Ratios of isoform transcriptional expression analyzed by Q-PCR**. Isoform median ratios are compared to Pan HLA-G ex5–6 isoform expression in healthy controls (solid line), patients with mild asthma (dashed line), and patients with severe asthma (dashed and dotted line) at day 0 (D0), D7, D14, and D21. Medians with range are described in Table S4 in Supplementary Material.

HLA-G isoform ratios, calculated in comparison with pan HLA-G ex5–6, showed that HLA-G1/-G5 were the most expressed isoforms in HC [9.81 (0.25–12.26)] and in JEG-3 [2.8 (2.10–16.11)] (Figure 2; Table S4 in Supplementary Material). It is noteworthy that HC displayed higher ratios for ex1–2 and HLA-G5/-G6 [5.23 (1.77–6.79) and 0.12 (0.03–1.38)] than JEG-3 [0.21 (0.05–0.52) and 0.04 (0.009–0.32)].

### HLA-G Isoform Correlations

Correlation of expression was investigated for each pair of isoforms in asthmatic patients and controls. Expression of pan HLA-G ex5–6, HLA-G1/-G4/-G5, and HLA-G1/-G5, all displayed significant positive correlation at D21 for HC, MA, and SA (Figure S2 in Supplementary Material). Therefore, these data were cumulated in association analyses and referred to as “cumulated pan-G1-G4-G5.”

Expression of ex1–2 of HLA-G displayed no correlation with any other HLA-G isoforms.

HLA-G2/-G6 showed a significant positive correlation with pan HLA-G ex5–6, HLA-G1/-G4/-G5, and HLA-G1/-G5 at D0 for HC and SA, but not for MA. HLA-G5/-G6 displayed a significant positive correlation with HLA-G1/-G4/-G5, HLA-G1/-G5, and HLA-G2/-G6 at D0 for HC.

### HBEC Derived from Asthmatic Patients Displayed Lower HLA-G Expression and Specific Isoform Ratios

**Figure 3:**
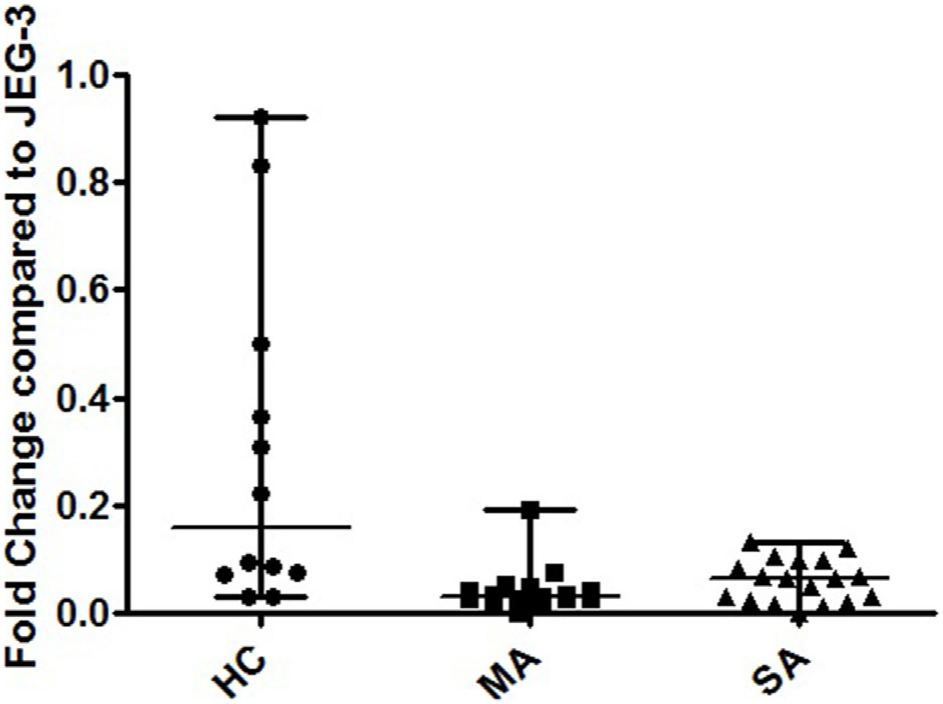
**Transcriptional expression analyzed by Q-PCR of cumulated HLA-G1/-G4/-G5, HLA-G1/-G5, and pan HLA-G Ex5–6 in human bronchial epithelium cells (HBEC) on day 21**. Fold change compared to JEG-3 in HBEC from healthy controls (HC) [0.156 (0.03–0.92)], patients with mild asthma (MA) [0.03 (0.0008–0.19)], and patients with severe asthma (SA) [0.06 (0.001–0.13)]. Kruskal–Wallis ANOVA performed on three modalities: *p* = 0.047. HC showed a significantly higher expression of cumulated pan-G1-G4-G5 than both MA and SA (*p* < 0.001 and *p* < 0.05, respectively; Mann–Whitney test).

HLA-G isoform expression and ratios displayed different patterns in fully re-differentiated HC, MA, and SA. At D21, HC showed a significantly higher expression of cumulated pan-G1-G4-G5 than both MA and SA [Kruskal–Wallis ANOVA performed on three modalities: *p* = 0.047, Mann–Whitney performed on two modalities HC vs. MA *p* < 0.001 and HC vs. SA *p* < 0.05; HC: 0.156 (0.03–0.92), MA: 0.03 (0.0008–0.19), SA: 0.06 (0.001–0.13); Figure 3].

At D21, SA displayed the highest ex1–2 (pan HLA-G) expression; however, this difference was not significant (Figure 1; Table S3 in Supplementary Material). Ex1–2 (pan HLA-G) showed the highest ratio compared to other isoforms for both MA [4.26 (1.03–13.94)] and SA [14.35 (1.47–22.93)], whereas in HC the highest ratio was for HLA-G1/-G5 [9.81 (0.25–12.26)] compared to SA [5.18 (0.57–11.30)] and MA [0.50 (0.26–8.46)]. HLA-G5/-G6 ratio in HC [0.12 (0.03–1.38)] was higher compared to MA [0.07 (0–3.07)], SA [0.04 (0.02–0.90)]. No difference was observed between HC, MA, and SA for HLA-G2/-G6. HLA-G3 had the lowest ratio among HLA-G isoforms with even an absence of signal for some patients. At D21, only SA expressed this isoform.

**Figure 4:**
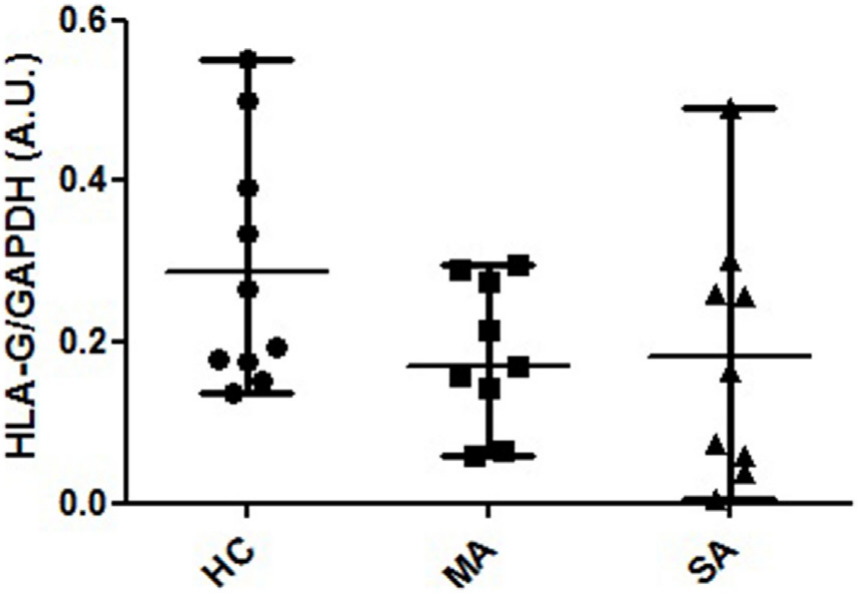
**HLA-G protein expression in human bronchial epithelium cell lysates from healthy controls (HC), patients with mild asthma (MA), and patients with severe asthma (SA)**. Semi-quantitative analysis (A.U., arbitrary units, mean and range) of Western Blot 4H84 staining normalized with GAPDH (mAb clone 9545) staining in HC [*N* = 10; 0.231 (0.137–0.552)], MA [*N* = 9; 0.186 (0.06–0.291)] and SA [*N* = 9; 0165 (0.004–0.492)]. The bands observed at 39 and 37 kDa may correspond to the biggest open reading frame from nucleotide 1 of HLA-G1 and HLA-G5 mRNA, respectively. Kruskal–Wallis ANOVA (HC, MA, SA) *p* = 0.214, non-significant; Mann–Whitney (HC vs. MA and SA) *p* = 0.088, non-significant.

Protein HLA-G expression was assessed in HC, MA, and SA. Alpha1 domain expression (4H84 antibody) was found to be non-significantly higher in HC compared to MA and SA [0.231 (0.137–0.552), 0.186 (0.06–0.291), 0.165 (0.004–0.492), respectively; Kruskal–Wallis ANOVA HC, MA, SA *p* = 0.214, non-significant; Mann–Whitney HC vs. MA and SA *p* = 0.088, non-significant; Figure 4; Table S5 and Figure S3 in Supplementary Material]. The bands observed at 39 and 37 kDa may correspond to the biggest open reading frame from nucleotide 1 of HLA-G1 and HLA-G5 mRNA, respectively.

### HBEC Derived from SA Patients Display Specific HLA-G Expression during Re-Differentiation

**Figure 5:**
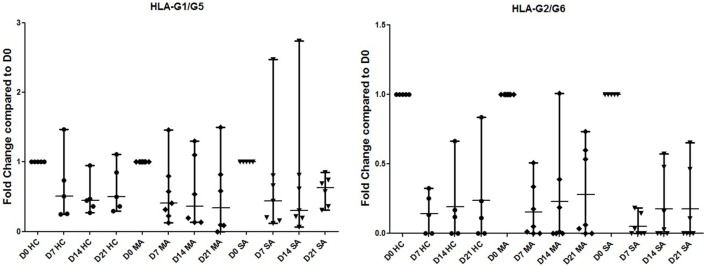
**Transcriptional expression analyzed by Q-PCR of HLA-G1/G5 and HLA-G2/G6 in healthy controls (HC), patients with mild asthma (MA), and patients with severe asthma (SA)**. Expression fold change during human bronchial epithelium cells re-differentiation is compared to D0. HLA-G1/-G5 expression is significantly higher at D0 for HC and MA, but not for SA (Kruskal–Wallis ANOVA: *p* = 0.033, *p* = 0.013, and *p* = 0.425, respectively). HLA-G2/-G6 expression was significantly lower at D0 for HC, MA, and SA (Kruskal–Wallis ANOVA: HC, MA, and SA *p* < 0.0001).

Differences in HLA-G isoform ratios during HBEC re-differentiation from D0 to D21 were observed between HC, MA, and SA (Figure 2; Table S4 in Supplementary Material). HC maintained the highest HLA-G1/-G5 ratio from D7. In SA, ex1–2 (pan HLA-G) showed an increased ratio, whereas it was stable in HC and MA from D7. When compared to D0, HLA-G1/-G5 expression decreased significantly at D7, D14, and D21 for HC (*p* = 0.033) and MA (*p* = 0.013), but not for SA (*p* = 0.425) (Figure 5). HLA-G2/-G6 expression decreased significantly on D7, D14, and D21 for HC, MA, and SA (*p* < 0.0001) (Figure 5). At D0, HLA-G3 was expressed by some individuals in HC (*N* = 4), MA (*N* = 3), and SA (*N* = 6), but the expression was only maintained in SA at D21 (*N* = 2). No significant variation was observed during re-differentiation for the other HLA-G isoforms.

### Asthmatic Patients Display Specific HLA-G Alleles

HLA-G*01:01 frequency was higher in HC (*N* = 10, Fq = 1) than both MA and SA (both *N* = 9, Fq = 0.64; χ^2^, *p* = 0.03). Asthmatic patients displayed HLA-G*01:04 (MA *N* = 2, Fq = 0.14; SA *N* = 1, Fq = 0.07) and HLA-G*01:06 (MA *N* = 3, Fq = 0.22; SA *N* = 4, Fq = 0.29). One MA patient carried an HLA-G*01:04 allele and an unreported allele (C/C at position 351 and C/T at position 800, respectively, excluded and included in the HLA-G*01:06 allele). HLA-G*01:06 allele frequency in MA and SA were significantly higher compared to healthy populations from South-East France (38) (χ^2^, respectively, *p* = 0.03 and *p* < 0.001). HC, MA, and SA showed equal frequencies for ex8 ins (HC *N* = 5, Fq = 0.50; both MA and SA *N* = 8, Fq = 0.57) and ex8 del.

### HLA-G Expression and Genetic Polymorphism

No significant difference was observed in HLA-G expression according to ex8 genotype. Ex8 del displayed a significant positive correlation with HLA-G1/-G4/-G5, HLA-G1/-G5, HLA-G3, and ex5–6 (pan HLA-G) at D21 in SA but not in HC and MA (Figure S2 in Supplementary Material). No correlation was observed between ex8 ins and any of the isoforms. Ex8 ins/del heterozygous individuals showed no correlation between both alleles expression.

Taking MA and SA together, cumulated pan-G1-G4-G5 showed a non-significantly higher median expression in HLA-G*01:04 individuals [0.53 (0.03–1.04)] than in HLA-G*01:01 individuals [0.05 (0.01–0.18)] and HLA-G*01:06 individuals [0.04 (0.02–0.07)].

### HLA-G Expression Is Not Modulated By IL-13 Stimulation

**Figure 6:**
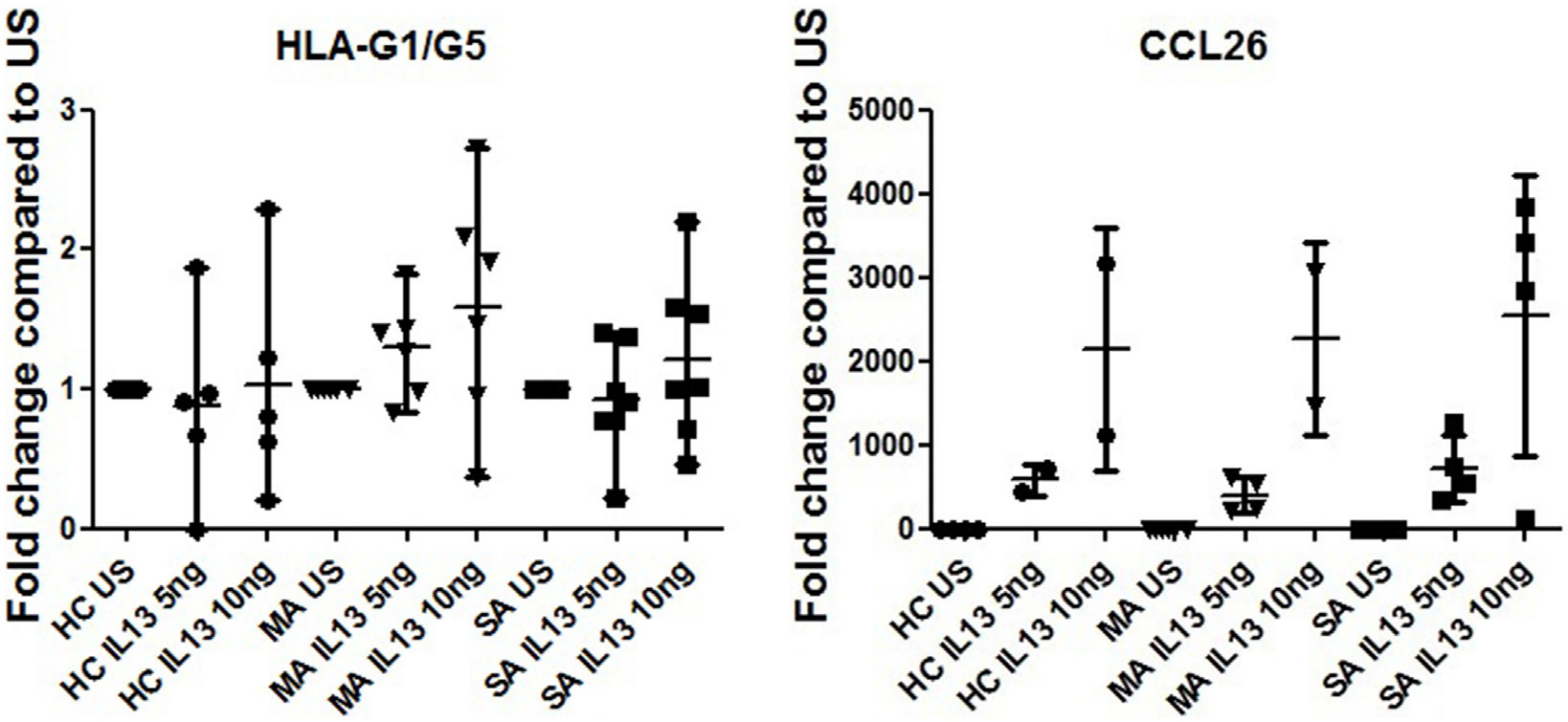
**HLA-G1/G5 and CCL26 (eotaxin-3) expression fold change at D21 in human bronchial epithelium cells (HBEC) stimulated with 5 and 10 ng of IL-13 from healthy controls, patients with mild asthma (MA), and patients with severe asthma (SA) compared to unstimulated samples (US)**. Kruskal–Wallis ANOVA performed on three modalities. IL13 stimulation had no effect on HLA-G expression in any of the HBEC tested. CCL26 (eotaxin-3) expression was significantly higher in stimulated compared to US (*p* < 0.001).

IL13 stimulation had no effect on HLA-G expression in any of the HBEC tested (Figure 6) or during the re-differentiation process (*p* = 0.419). IL-13 stimulation was, however, effective, as shown by CCL26 (eotaxin-3) expression, which was significantly higher in stimulated compared to unstimulated samples (*p* < 0.0001).

## Discussion

Inflammation of the airways and dramatic bronchial epithelium structural remodeling are features of asthma. This disease includes various phenotypes which are unequally controlled by therapeutics, mostly based on the suppression of immune cell activation. Thus, attempts are made to find alternative therapies in order to manage non-responding patients, most of whom are severe asthmatic. Given that the immunotolerant HLA-G molecule is well-known for its implication in various inflammatory conditions, a more comprehensive analysis of its local bronchial expression in asthmatic patients could bring this molecule into the limelight as a new therapeutical strategy.

The main objectives of this study were to show that HLA-G isoform expression was impaired in an asthmatic context and to confirm that HLA-G isoform expression was not affected by a typical T2 cytokine, namely IL-13.

The present study is the first to have investigated into the expression of all of the HLA-G mRNA isoforms. All HLA-G isoforms were expressed in fully re-differentiated HBEC from HC, MA, and SA. HLA-G mRNA expression was significantly reduced in both MA and SA compared to HC. HLA-G mRNA expression was not affected by IL-13. Furthermore, genotyping results showed that both MA and SA displayed higher HLA-G*01:04 and *01:06 allelic frequencies.

No currently available commercial tools allow the quantification of the different HLA-G protein isoforms, the 4H84 antibody recognizes all HLA-G isoforms. In our study, HLA-G mRNA isoform expression was investigated by the highly sensitive Real-time TaqMan^®^ PCR technology in HBEC–ALI from both asthmatic patients and healthy donors. HBEC in ALI culture maintain morphological features and disease markers associated with mild and severe asthma (6). Most importantly, this *ex vivo* model allows epithelial analysis without any immune cells and cytokines known to influence HLA-G expression [for review, see Ref. (18)] and represents a most suitable model to investigate into the specific signature of normal and asthmatic HBEC HLA-G expression. Furthermore, ALI monoculture allows dynamic analysis of re-differentiation for 21 days.

We observed a significantly impaired HLA-G mRNA expression of cumulated pan-G1-G4-G5 in both MA and SA compared to HC in a fully re-differentiated epithelium. This lower expression could be associated to the specific allelic pattern displayed by asthmatic patients, as MA and SA showed a significantly lower frequency of HLA-G*01:01 compared to HC. HLA-G*01:06 allele was significantly higher in both MA and SA compared to the healthy population from South-East France (38). These results are in accordance with HLA-G being defined as a potential asthma susceptibility gene by fine-mapping and positional candidate study (11) and with previous studies showing a negative effect of HLA-G*01:04 and HLA-G*01:06 in bronchial epithelium pathological situations (39). These alleles could have a negative influence on bronchial epithelium capability of resolving inflammation either because they code for a less functional protein or, more likely, because they express less protein. Whether these coding alleles are associated with an impaired protein or with differential quantitative expression needs to be further investigated; however, former studies have shown that HLA-G*01:04 and HLA-G*01:06 are associated with specific SNPs in regulatory regions with an impact on their expression (38, 40).

The genotype ex8 ins/del did not have any effect on HLA-G mRNA expression in HC, MA, or SA, in agreement with Zheng et al. who showed no relationship between ex8 ins/del status and sHLA-G plasma levels in allergic asthma in childhood (13). Former studies showed that the ex8 deletion confers a higher soluble/membrane-bound HLA-G expression ratio whereas the ex8 insertion is associated with higher degree of mRNA stability, increased membrane-bound HLA-G expression, and more efficient inhibition of NK cytotoxicity (26). Here, we found that expression of isoforms bearing the ex8 deletion only correlated with cumulated pan-G1-G4-G5 in SA. Thus, SA might also display a lower HLA-G membrane/soluble ratio and a less efficient immune cell inhibition capacity than in MA and HC.

The influence of the other polymorphisms in regulatory regions, such as SNP −964 and +3,142 associated with asthma (11, 41), could not be explored here as genomic DNA was not available. SNPs in both 5′ UTR and 3′ UTR are in linkage disequilibrium with coding alleles and are associated with HLA-G differential expression (38, 40), so they deserve to be addressed in a specific asthmatic cohort genetic study. Indeed, high resolution genetic analysis of both alleles and regulatory regions could shed some light on the HLA-G expression heterogeneity observed here.

Previous studies supported, however, a higher level of soluble HLA-G protein and local expression in asthmatic individuals compared to HCs (12, 13). Here, no significant difference between asthmatic patients and healthy individuals could be distinguished by western blot analysis, and not all protein isoforms were observed. The quantity of translated protein may be too low to be detected by western blot, a hypothesis supported by Q-PCR isoforms ratio results, or these mRNA isoforms may not be translated, raising the question of their functional relevance. Furthermore, HLA-G mRNA and soluble protein expression are not always correlated, HLA-G expression can be highly heterogeneous according to cell type, and the proteins can be stored in the cytoplasm (28, 42). Some authors also hypothesized that at steady state, soluble HLA-G can be cleared from the organism at a certain rate and that some individuals can present higher soluble HLA-G blood levels although they produce a lower quantity of mRNA (26). Moreover, soluble HLA-G expression, not correlated to membrane-bound expression (43), can be expressed by different and/or distant cells from the bronchial epithelium (44).

MA and SA displayed specific alternatively spliced HLA-G isoform expression, correlation, and ratios; however, little is known about the exact function of these alternative isoforms. HLA-G2, -G4, and -G6 have been reported to display tolerogenic activity (20), and conflicting results have been reported concerning HLA-G3, possibly due to chaperone proteins specific to the cell lines used (20, 24).

It is notable that the expression of exons 1 and 2, coding for the signal peptide, was higher in all HBEC than in JEG-3, SA displaying the highest ratio. Expression of exons 1 and 2 were not correlated with any of the other isoforms. No published data targeting exon 1 have also analyzed other exons (45) with which to confront our results. Differential expression of exons 1 and 2 could specifically enhance HLA-E expression to resolve inflammation; over-expression of HLA-E has been previously reported in tumor cells in colorectal cancer and was associated with the inhibition of NK or CD94+/NKG2A+/CD8+ T cells infiltrating tumor tissue (46).

HLA-G1/-G5 and HLA-G2/-G6 expression were the highest in undifferentiated HBEC, at D0, for HC, in accordance with HLA-G being expressed by undifferentiated cells as reported in mesenchymal stem cells (47). These results could support the involvement of HLA-G in epithelial proliferation and differentiation potentials (16, 17).

SA displayed specific HLA-G isoform expression starting from the earlier stages of re-differentiation through to D21. Thus, both bronchial epithelium structural remodeling and impairment in inflammation resolution observed in asthma could be the result of HBEC intrinsic defects, embodied here by HLA-G isoform expression, rather than immune cell dysfunction.

The IL-13 activation pathway has been reported to act directly on airway epithelium inducing epithelial cell proliferation and differentiation (48). Here, IL-13 induced no modification of HLA-G isoform expression in any of the ALI cultures, extending the results of White et al. to asthmatic cells (14). Thus, even though IL-13 has a striking effect on airway epithelium, it does not affect HLA-G expression.

In conclusion, our study promotes HBEC in ALI culture as a suitable model for HLA-G expression analysis. We showed that HBEC obtained and cultivated in ALI from SA patients displayed a specific HLA-G isoform expression profile compared to those from MA and HC. Many mechanisms could lead to an impairment of HLA-G expression resulting from local asthmatic micro-environment specificities and subordinated by HLA-G genetic polymorphisms: transcriptional factors (18, 49), miRNA (41, 45, 50), epigenetic modifications (51), or splicing factors (52).

Whatever the inner processes of HLA-G regulation in HBEC, differential HLA-G isoform expression could be responsible for the loss of inflammation control and for epithelium structural remodeling in asthmatic patients. Therefore, this molecule might be an interesting alternative candidate to target for asthmatic patients not adequately responding to treatments focused on immune cell effectors. Many questions remain, however, in particular, deciphering the mechanism(s) responsible for differential HLA-G isoform expression, identifying the epithelial cell type(s) expressing HLA-G, and elucidating the function of each isoform and their actual receptor, if any, in the local bronchial micro-environment.

## Author Contributions

Conception or design of the work: DP, CP, PC, DG, and JDC. Acquisition, analysis and interpretation of data: FC, CG, DP, PR, JC, CP, PC, DG, and JDC. Drafting the work or revising it critically for important intellectual content: FC, CG, DP, PR, JC, CP, PC, DG, and JDC. Final approval of the version to be published: FC, CG, DP, PR, JC, CP, PC, DG, and JDC. Agreement to be accountable for all aspects of the work in ensuring that questions related to the accuracy or integrity of any part of the work are appropriately investigated and resolved: FC, CG, DP, PR, JC, CP, PC, DG, and JDC.

## Conflict of Interest Statement

The authors declare that the research was conducted in the absence of any commercial or financial relationships that could be construed as a potential conflict of interest.

## Supplementary Material

Table S1 **Primer and probe characteristics used for HLA-G allele typing (primers combination F1/R3, F2/R4, and F3R5) and for HLA-G Q-PCR analyses**.

Table S2 **Plasmid characteristics used for HLA-G Q-PCR efficiency analyses**.

Table S3 **Transcriptional HLA-G expression analyzed by Q-PCR**. Median fold change with range compared to JEG-3 in healthy controls (HC), patients with mild asthma (MA), and patients with severe asthma (SA) at day 0 (D0), D7, D14, and D21, except for HLA-G Pan ex8 del calibrated with human bronchial epithelium cells HC.

Table S4 **Transcriptional HLA-G analyzed by Q-PCR**. Isoform median and range ratios are compared to Pan HLA-G ex5–6 expression in healthy controls, patients with mild asthma (MA), and patients with severe asthma (SA) at day 0 (D0), D7, D14, and D21. ^†^Only one point available.

Table S5 **HLA-G protein expression in human bronchial epithelium cell lysates from healthy controls (HC), patients with mild asthma (MA), and patients with severe asthma (SA)**. Semi-quantitative analysis (A.U., absorbance units, mean and range) of Western Blot with 4H84, staining normalized with GAPDH (mAb clone 9545). Kruskal–Wallis ANOVA (HC, MA, SA) *p* = 0.214, non-significant; Mann–Whitney (HC vs. MA and SA) *p* = 0.088, non-significant.

Figure S1 **Illustration of the alternatively spliced HLA-G isoforms**. The nine primers/probe used in the study are illustrated by arrows, lines and specific colors.

Figure S2 **Correlations between each HLA-G isoform at D0 and D21 for healthy controls, patients with mild asthma (MA), and patients with severe asthma (SA)**. Statistical significance is highlighted (light gray: *p* < 0.05 and gray: *p* < 0.001). *Correlations performed only for heterozygous ins/del individuals. ^†^Number of data not sufficient to perform correlation test.

Figure S3 **Protein HLA-G expression in human bronchial epithelium cell from healthy controls (*N* = 4), patients with mild asthma (MA, *N* = 4), and patients with severe asthma (SA, *N* = 4)**. Representative image of HLA-G isoforms detection by western blot with 4H84 mAb.

## Abbreviations

PBMC, peripheral blood mononuclear cells; ALI, air–liquid interface; FEV1, forced expiratory volume in 1 s; GERD, gastroesophageal reflux disease; HBEC, human bronchial epithelial cells; ICS, inhaled corticosteroid; LAβ2, long-acting β2 agonist; SAβ2, short acting β2 agonist; OCS, oral corticosteroid.
