# Supplementary figures and images for: Bronchial Epithelial Cells from Asthmatic Patients Display Less Functional HLA-G Isoform Expression
Source: Front Immunol. 2017 Jan 23;8:6. doi: 10.3389/fimmu.2017.00006 (PMC5333864; doi:10.3389/fimmu.2017.00006)

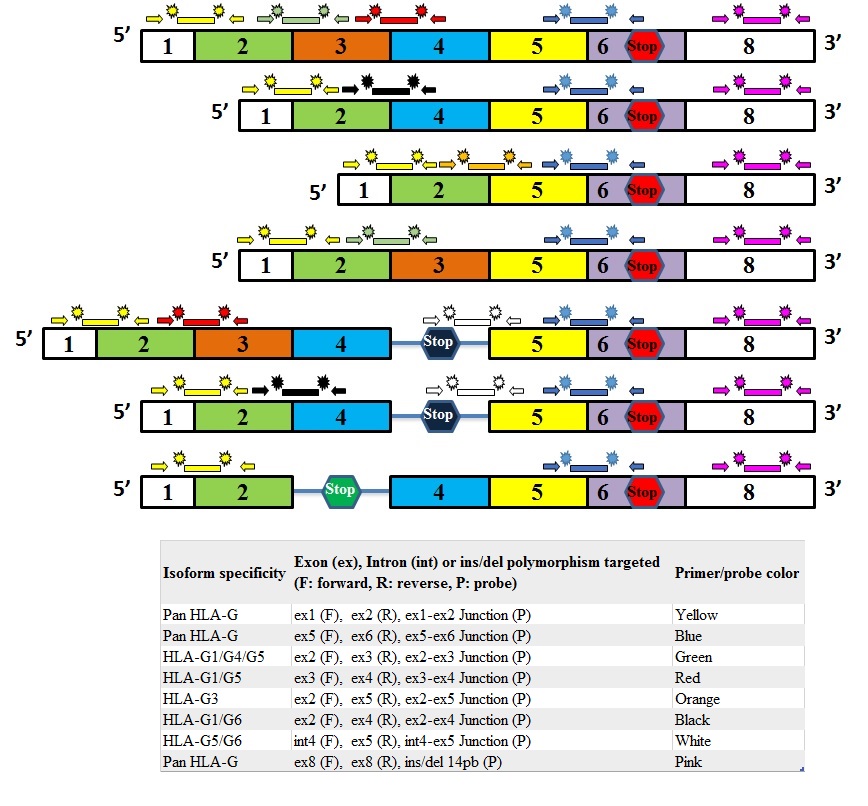

Supplement: Figure S1 — Illustration of the alternatively spliced HLA-G isoforms. The nine primers/probe used in the study are illustrated by arrows, lines and specific colors. [file image_1.jpeg]

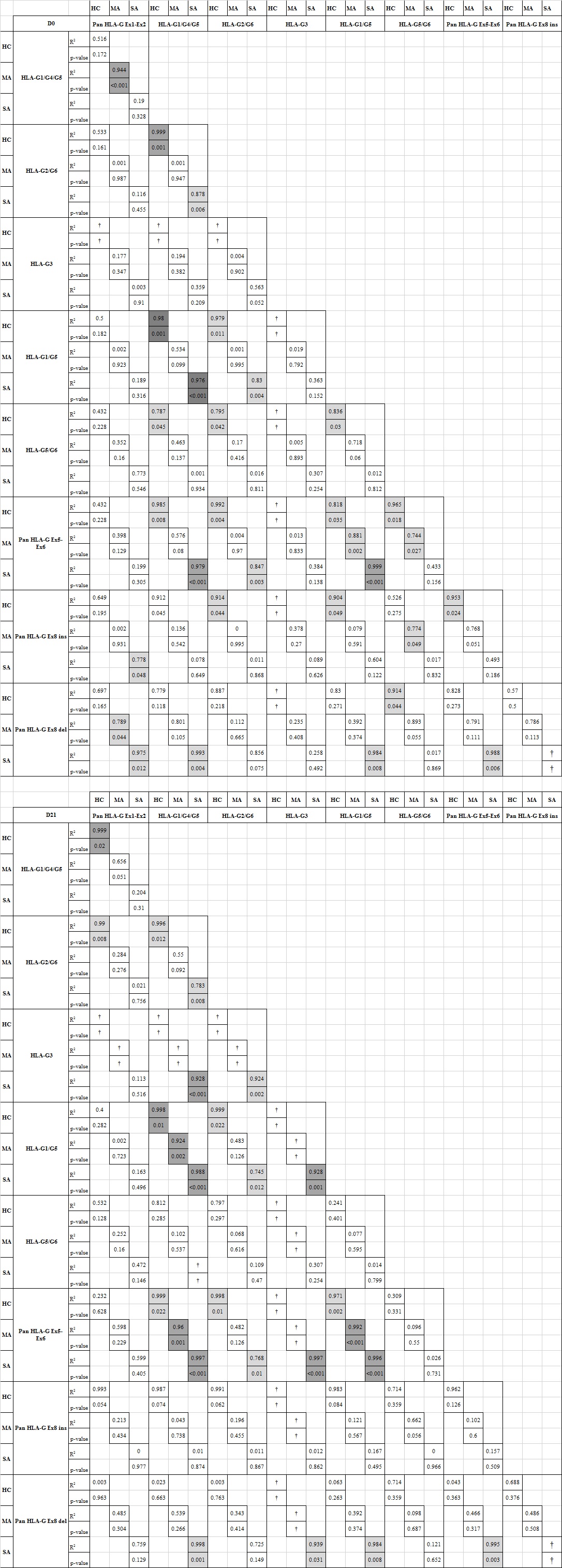

Supplement: Figure S2 — Correlations between each HLA-G isoform at D0 and D21 for healthy controls, patients with mild asthma (MA), and patients with severe asthma (SA). Statistical significance is highlighted (light gray: p < 0.05 and gray: p < 0.001). *Correlations performed only for heterozygous ins/del individuals. †Number of data not sufficient to perform correlation test. [file image_2.jpeg]

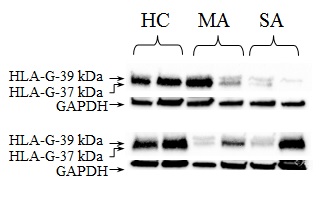

Supplement: Figure S3 — Protein HLA-G expression in human bronchial epithelium cell from healthy controls (N = 4), patients with mild asthma (MA, N = 4), and patients with severe asthma (SA, N = 4). Representative image of HLA-G isoforms detection by western blot with 4H84 mAb. [file image_3.jpeg]
